# Supplementary material for: Chitosan-G-Glycidyl Methacrylate/Au Nanocomposites Promote Accelerated Skin Wound Healing
Source: Pharmaceutics. 2022 Sep 2;14(9):1855. doi: 10.3390/pharmaceutics14091855 (PMC9505090; doi:10.3390/pharmaceutics14091855)
Supplement: Supplementary file 1 [file pharmaceutics-14-01855-s001.zip › pharmaceutics-1858975-supplementary.pdf]

Table S1. Valuation diagram of wounds: 1= recover, 4 = limited recover. According with score the wound can be classified in four types.

Score	1	2	3	4
Aspect	Erythematous	Blushed	Yellow	Necrotic
Lesion extension	0-1 Cm	>1-3cm	>3-6 cm	>6 cm
Lesion deep	0	<1 cm	1-3 Cm	>3 cm
Exudate quantity	Absent	Insufficient	Moderate	Abundant
Exudate quality	Without exudate	Serous	Muddy	Purulent
Necrotic tissue	Absent	< 25	25-50 %	>50 %
Granular tissue	100-75%	< 75-50 %	< 50-25 %	< 25 %
Edema	Absent	+	++	+++
Pain	0-1	2-3	4-6	7-10
Surrounding skin	Healthy	Flaking	Erythematous	Macerated

**Classification of wounds by score**	
Type 1	10 to 15 points
Type 2	16 to 21
Type 3	22 to 27
Type 4	28 to 40
